# Metabolomics investigation of recombinant mTNFα production in *Streptomyces lividans*

**DOI:** 10.1186/s12934-015-0350-1

**Published:** 2015-10-09

**Authors:** Howbeer Muhamadali, Yun Xu, David I. Ellis, Drupad K. Trivedi, Nicholas J. W. Rattray, Kristel Bernaerts, Royston Goodacre

**Affiliations:** School of Chemistry, Manchester Institute of Biotechnology, University of Manchester, Manchester, UK; Bio- and Chemical Systems Technology, Reactor Engineering and Safety, Department of Chemical Engineering, KU Leuven (University of Leuven), Leuven Chem&Tech, Celestijnenlaan 200F (bus 2424), 3001 Leuven, Belgium

**Keywords:** *Streptomyces*, FT-IR spectroscopy, GC–MS, Metabolic fingerprint, Biotechnology, Metabolomics, Recombinant protein production, Metabolic profile, Metabolic footprint, Synthetic biology

## Abstract

**Background:**

Whilst undergoing differentiation, *Streptomyces* produce a large quantity of hydrolytic enzymes and secondary metabolites, and it is this very ability that has focussed increasing interest on the use of these bacteria as hosts for the production of various heterologous proteins. However, within this genus, the exploration and understanding of the metabolic burden associated with such bio-products has only just begun. In this study our overall aim was to apply metabolomics approaches as tools to get a glimpse of the metabolic alterations within *S.lividans* TK24 when this industrially relevant microbe is producing recombinant murine tumour necrosis factor alpha (mTNFα), in comparison to wild type and empty (non-recombinant protein containing) plasmid-carrying strains as controls.

**Results:**

Whilst growth profiles of all strains demonstrated comparable trends, principal component-discriminant function analysis of Fourier transform infrared (FT-IR) spectral data, showed clear separation of wild type from empty plasmid and mTNFα-producing strains, throughout the time course of incubation. Analysis of intra- and extra-cellular metabolic profiles using gas chromatography–mass spectrometry (GC–MS) displayed similar trends to the FT-IR data. Although the strain carrying the empty plasmid demonstrated metabolic changes due to the maintenance of the plasmid, the metabolic behaviour of the recombinant mTNFα-producing strain appeared to be the most significantly affected. GC–MS results also demonstrated a significant overflow of several organic acids (pyruvate, 2-ketoglutarate and propanoate) and sugars (xylitol, mannose and fructose) in the mTNFα-producing strain.

**Conclusion:**

The results obtained in this study have clearly demonstrated the metabolic impacts of producing mTNFα in *S. lividans* TK24, while displaying profound metabolic effects of harbouring the empty PIJ486 plasmid. In addition, the level of mTNFα produced in this study, further highlights the key role of media composition towards the efficiency of a bioprocess and metabolic behaviour of the host cells, which directly influences the yield of the recombinant product.

**Electronic supplementary material:**

The online version of this article (doi:10.1186/s12934-015-0350-1) contains supplementary material, which is available to authorized users.

## Background

Usually found inhabiting soil and decaying vegetation, *Streptomyces* are very significant and highly important bacteria which are probably the most widely known, as well as widely studied, genus of the phylum Actinobacteria. These Gram-positive spore-producing filamentous bacteria comprise over 500 species [1] and have a complex secondary metabolism, as a consequence of which they are the largest microbial producer of antibiotics [2] providing the majority of antibiotics currently in use.

The complex, and frankly fascinating, metabolism of streptomycetes (in addition to a complex morphological life-cycle) is due to their unusually large bacterial genome [3, 4]. Whilst undergoing differentiation, leading to sporulation, a large quantity of hydrolytic enzymes and secondary metabolites are known to be secreted [5], and it is perhaps this very ability that has focused increasing interest on the use of these bacteria as hosts for the production of various heterologous proteins. These include the production of antifungal compounds and antibacterial agents, in addition to anti-parasitic [6] and anticancer drugs [7, 8]. *Streptomyces* spp. are therefore invaluable to human health with the possibility for yet more antibiotics still to be discovered with further developments in screening of these, and other species of bacteria [9], as well as the analyses of cryptic gene clusters [10]. Although, the most popular and intensely studied species of the genus is *S.* *coelicolor*, *S.* *lividans* is the organism of choice within the genus for heterologous protein production purposes. This is mainly due to *S.* *lividans* exhibiting less extracellular proteolytic activity and also a lack of the strong restriction system of *S. coelicolor* [11–13]. Some important enzymes, as well as recombinant proteins are produced by *Streptomyces*, including the murine tumour necrosis factor alpha (mTNFα) [12, 14, 15] and human glucagon [16]. For an overview of recombinant protein production and some recent examples of heterologous (mammalian and microbial) proteins produced using *S.* *lividans* as the host organism, as well as how systems biology (including metabolomics) approaches can be developed to improve protein production, the reader is directed to the following excellent reviews [17, 18].

The bioprocesses associated with the production of foreign proteins [19, 20], as well as harbouring recombinant plasmids within cells [21], have the potential to impose some form of metabolic stress to these microorganisms. We consider that metabolomics approaches may well provide additional information, and a far better understanding of any metabolic burden from within a cell resulting from protein production. This has the potential to assist directly in the optimisation of the overall bioprocessing methodology, which would ideally result in the enhancement of bioproduct formation [22]. Using an array of analytical platforms, metabolomics approaches not only have the ability to quantify metabolites under defined sets of cellular states and at multiple time points, but also allows for the dynamics of any form of abiotic or biotic perturbation to be accurately assessed [23, 24]. Not surprisingly, the field of metabolomics has become increasingly popular for several reasons, which are detailed in far more depth elsewhere [25–28] but include the ability for the precision measurement of multiple metabolites accurately and directly from complex biological systems. In addition, as the metabolome is considered a downstream process to the genome, transcriptome and proteome it may also provide a clearer image of an organism’s phenotype [29], which will be measurably affected by any perturbation in these organisms.

Here, we investigate the metabolic effects of recombinant mTNFα production on *S. lividans* grown in a defined medium with glucose as the main carbon source and aspartate as the nitrogen source. As the future direction of this study is to employ fluxomics strategies to explore the contribution of carbon and nitrogen sources towards biomass and recombinant mTNFα production in *S. lividans*, and to examine the contribution of aspartate towards mTNFα production further, as suggested by D’Huys and colleagues, a defined medium was formulated.

Fourier transform infrared (FT-IR) spectroscopy was employed as a metabolic fingerprinting tool to examine the overall phenotypic changes in the biochemical composition of the cells, while gas chromatography–mass spectrometry (GC–MS) was used to identify the significant metabolites in the medium (metabolic footprinting) as well those recovered from cell extracts (metabolic profiling).

FT-IR spectroscopy is a rapid, highly reproducible, non-destructive, high-throughput screening tool well established in metabolomics [30, 31] and microbiology [32–34], as well as many other areas [35]. GC–MS is considered as one of the gold standard approaches for metabolic profiling and chemical characterization of metabolites [36–38]. Our overall aim being to use metabolomics approaches as tools to elucidate the complex metabolic processes and mechanisms further within recombinant protein producing strain of *S. lividans*, which may then in turn aid the further development, optimisation and enhancement of recombinant protein production in these highly important and industrially/clinically relevant species of bacteria.

## Results and discussion

Although a lot is known about the life cycle [39], protein secretion pathways [40, 41] and metabolism [42] of streptomycetes, there is very little known about the impact of heterologous protein production on bacterial metabolism and its subsequent effects on the performance and physiology of the organism itself. Recently, several studies have attempted to study this metabolic impact through applying different approaches including: amino acid profiling [43], metabolic flux analysis [44] and metabolic profiling approaches [45]. The current study is aimed at investigating the impact of heterologous mTNFα production on the metabolite pools and growth behaviour of *S. lividans* TK24 grown in a defined minimal medium using GC–MS as metabolic profiling and footprinting approaches, whilst employing FT-IR as a rapid metabolic fingerprinting strategy.

### Growth profile and mTNFα production

**Fig. 1:**
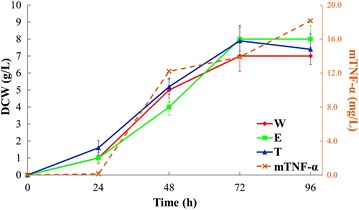
Growth profiles (DCW) of different *S. lividans* TK24 strains grown on minimal medium, wild (*W*, *red line*), empty plasmid containing (*E*, *green line*) and protein producing (*T*, *blue line*) strains. *mTNFα* level produced by the protein producing strain was quantified by ELISA (*orange dashed line*). DCW measurements are presented as means of eight biological replicates for each strain. *Errors bars* indicate standard deviations. The ELISA results are presented as means of three biological replicates

The growth behaviour of all three *S. lividans* strains in NMMP was monitored by measuring the dry cell weight (DCW) of each strain at different time points during the incubation period. Figure 1 displays similar growth behaviour for all three strains under the examined conditions. The mTNFα secreted or excreted into the medium was monitored by enzyme-linked immunosorbent assay (ELISA) and displayed an increasing trend with time, reaching the maximum concentration of 18.1 mg/L after 96 h of incubation. Although the concentration of mTNFα in the medium generally increased with the increasing level of biomass (Fig. 1), its production rate during the first 24 h of growth was significantly lower than the subsequent time points. In addition, after 72 h when cells reached the stationary phase, even though the biomass remained at a constant level, mTNFα concentration continued to increase.

D’Huys and colleagues [43] also reported a slower mTNFα production during initial growth followed by an increased production rate that continued through the stationary phase, which is in agreement with our findings. These authors also suggested that although the supplemented aspartate and glutamate in their study supported biomass production, it did not seem to contribute significantly towards protein (mTNFα) synthesis. Our DCW and mTNFα measurements are in agreement with the above findings. In this study aspartate has been used as the sole nitrogen source and the mTNFα levels detected (18.1 mg/L) were significantly lower than those reported by D’Huys and colleagues (~150 mg/L). The maximum biomass yield we obtained using aspartate as the nitrogen source was 8 g/L (Fig. 1) compared to ~5 g/L when D’Huys and co-workers [43] used ammonium sulfate as the nitrogen source. These findings lend support to the claim that aspartate is used as nitrogen and carbon sources supporting biomass production, however it does not contribute significantly towards mTNFα production, hence all three strains in this study displayed comparable growth behaviour.

### FT-IR fingerprint analysis

**Fig. 2:**
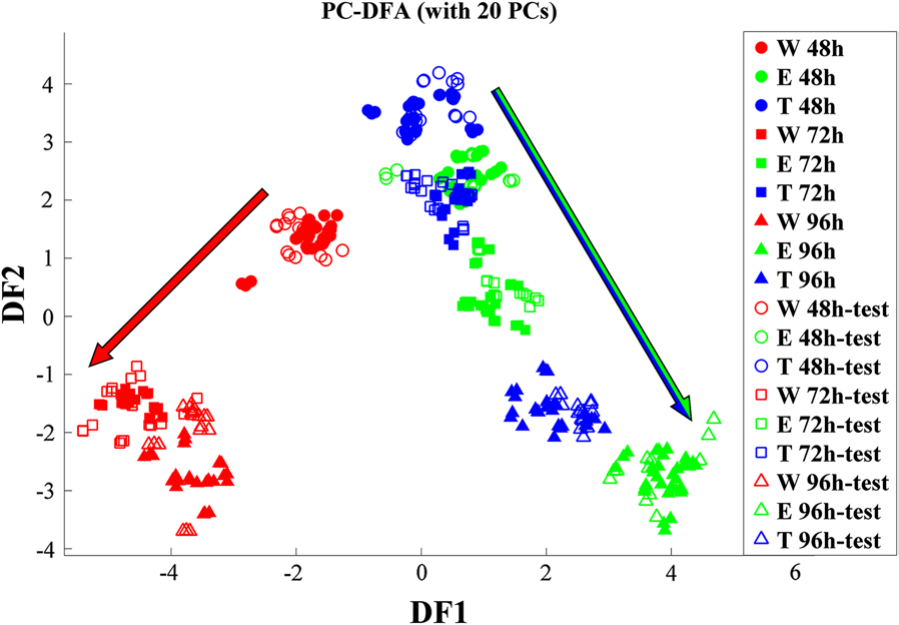
PC-DFA results of FT-IR spectra collected from different *S. lividans* TK24 strains at various time points during growth on minimal medium. Eight biological replicates and three technical replicates were used to generate the model which accounted for 99.8 % of the variance (20 PCs). *Different colours* represent different strains [wild (*W*, *red*), empty plasmid containing (*E*, *green*) and protein producing (*T*, *blue*) strains]. *Empty symbols* represent the projected validation data, and time points are displayed as different symbols (48 h *circle*, 72 h *square* and 96 h *triangle*). *Coloured arrows* represent the direction of separation according to incubation time

FT-IR spectroscopy was employed as a metabolic fingerprinting approach to examine the overall changes in the biochemical composition of the three *S. lividans* strains during growth. Samples taken after 24 h of incubation were excluded from the FT-IR analysis due to insufficient biomass yield. FT-IR spectral data collected from the following three time points (48, 72 and 96 h) were pre-processed as described in the “Methods” section and subsequently analysed by PC-DFA. The resultant ordination scores plot (Fig. 2) displayed a gradual separation between the three strains, due to phenotypic changes, with increasing incubation time. Figure 2 displays the separation of the wild type (W) from the empty plasmid-carrying (E) and mTNF-producing (T) strains according to discriminant function 1 (DF1), whilst DF2 separated the samples based on the incubation time (growth phase).

The principal components-discriminant function analysis (PC-DFA) results (Fig. 2) displayed tighter clustering of the empty plasmid (E 48 h) with mTNFα-producing strain (T 48 h) at 48 h of growth, after which these strains also started to separate at later time points (72 and 96 h). This separation could be a reflection of the recombinant mTNFα production on the mTNFα-producing strain, which could be enhanced with increasing incubation time and the depletion of nutritional resources in the medium.

### Metabolic profiles and footprints

**Fig. 3:**
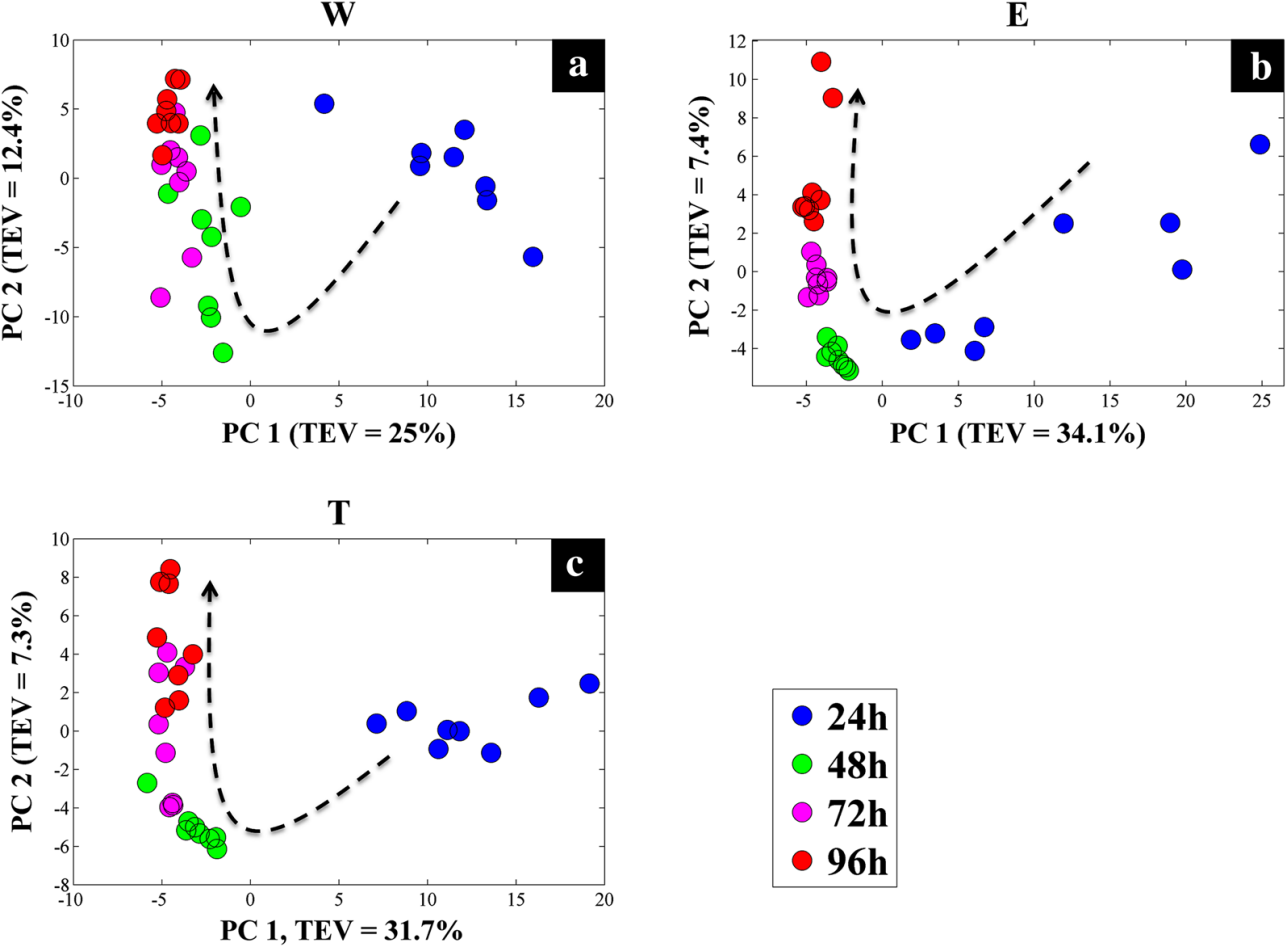
CPCA-W *scores plots* of the strain-blocked GC–MS metabolic profiles data. The *scores plots* for each of the strain-blocked data are presented on **a–c** *plots*, where samples taken at separate time points are presented by *different coloured circles*. The *dashed arrows* display the direction of the separation according to incubation time. *Different letters on each plot* indicate the *S. lividans* strains; wild (*W*), empty plasmid (*E*), and mTNF-producing (*T*)

GC–MS is one the most established techniques in the field of metabolomics due to its many advantages, and is considered as one of the gold standard technologies. In this study, the intracellular metabolic profiles (cellular extracts or endo-metabolome) and extracellular metabolic footprints (spent medium or exo-metabolome) of three different *S.* *lividans* strains were analysed by GC–MS. The normalized peak areas of the detected metabolites were subjected to weighted-consensus principal component analysis (CPCA-W) to study and compare the metabolic behaviour of mTNFα-producing and non-producing *S.* *lividans* strains at different time points. Initially the GC–MS dataset containing the metabolic profiles of all the samples was arranged into three blocks based on the three different *S. lividans* strains (strain-blocked), to compare the metabolic behaviour of each strain individually with respect to incubation time (Fig. 3).

**Fig. 4:**
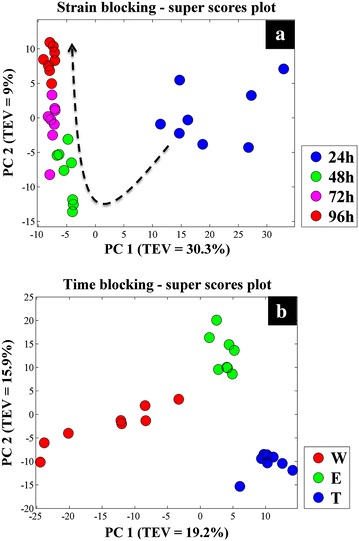
CPCA-W super *scores plots* of GC–MS metabolic profiles. **a** Super *scores plot* of the strain-blocked model, *different coloured symbols* indicate sampling time. **b** Super *scores plot* of the time-blocked model, where *coloured symbols* represent different *S. lividans* strains

Block-scores plots of the wild type data-block (Fig. 3a) demonstrated a clear separation between samples taken at 24 h of incubation and samples from the following time points (48, 72 and 96 h) according to principal component 1 (PC1) which accounted for 25 % of the total explained variance (TEV). However, the overall clustering pattern of the wild type data-block indicated a gradual time-dependant change in the metabolic profiles of the cells, as highlighted by the dashed arrow (Fig. 3a). Scores plots of the empty plasmid (Fig. 3b) and mTNFα-producing (Fig. 3c) strains followed the same clustering pattern, demonstrating the clear separation of the 24 h samples from the remaining samples according to PC1, whilst exhibiting a similar time-dependent clustering pattern. Super scores plot of the strain-blocked model (Fig. 4a) also exhibited this time-dependant trend, further confirming the above findings.

**Fig. 5:**
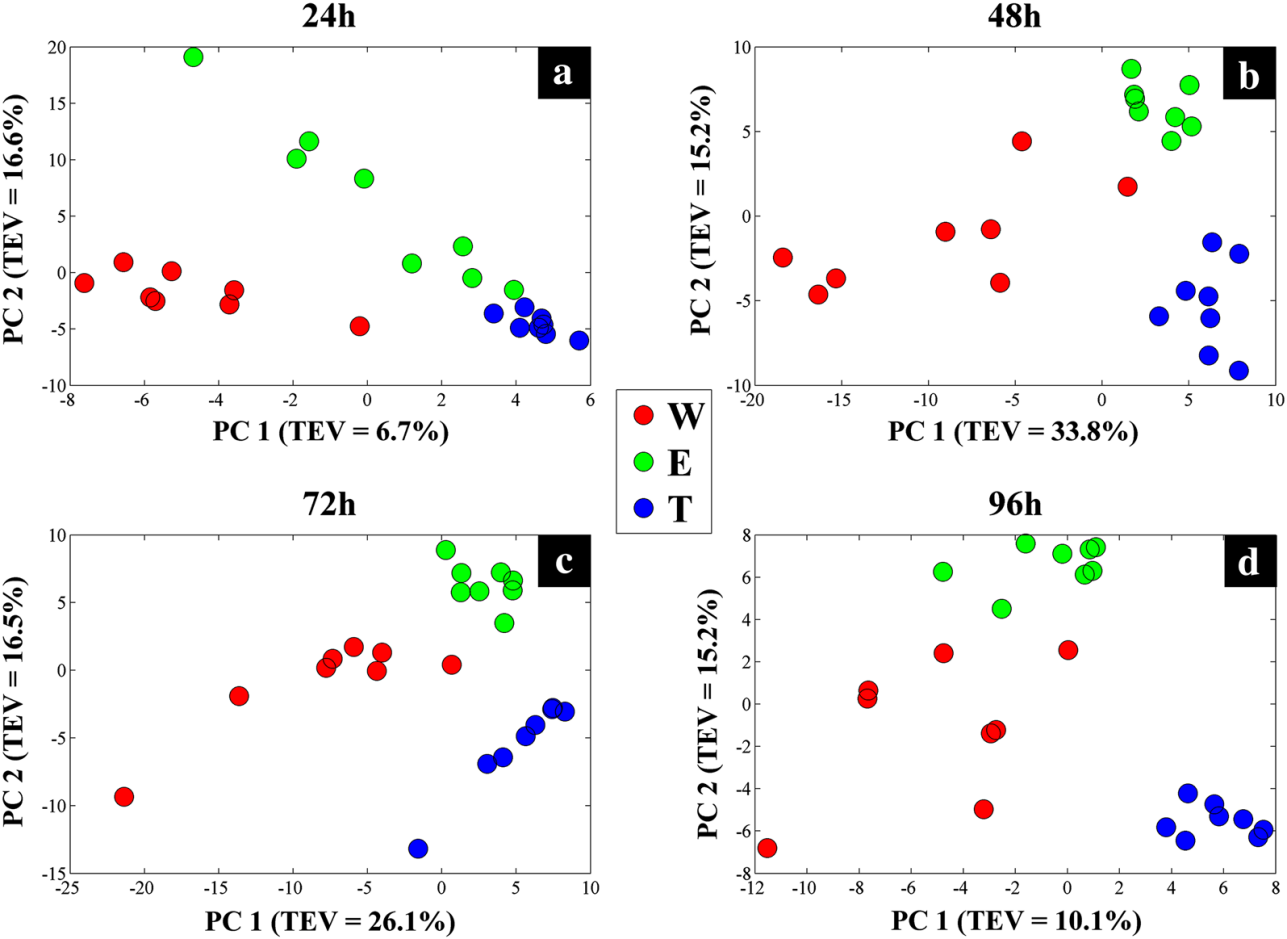
CPCA-W *scores plots* of the time-blocked GC–MS metabolic profiles data. The *scores plots* for each of the time-blocked data are presented on **a**–**d** plots, where *different coloured circles* present different *S. lividans* strains

In order to identify the similarities and differences in the metabolic profiles of the three strains at each time point individually, the dataset was rearranged into four blocks (time-blocked) based on the four sampling time points (24, 48, 72 and 96 h). The scores plot of the 24 h data-block (Fig. 5a) exhibited a clear separation between the three strains. Whilst the scores plots of the following time points exhibited similar clustering patterns (Fig. 5b–d), the separation increased with time. The strain most affected by incubation time appeared to be the mTNFα-producing strain (T), as it clustered further away from the two other strains. Super scores plot of the time-blocked model (Fig. 4b) was in agreement with the above findings as it also displayed the separation of all three strains.

CPCA-W results of the GC–MS footprint dataset (Additional file 1: Figure S1) displayed similar results to the metabolic profiles, where strain-blocked scores plots demonstrated a gradual time-dependent change in the metabolic footprint of each strain, which was in agreement with its corresponding super scores plot (Additional file 1: Figure S2a). Furthermore, the footprint time-blocked dataset displayed a tight clustering of the footprint data in the 24 h data-block (Additional file 1: Figure S3a) followed by a gradual separation in the following time-blocks (Additional file 1: Figure S3b–d). Super scores plot of the time-blocked footprint data set (Additional file 1: Figure S2b) also exhibited a clear separation between the mTNFα-producing (T) and non-producing strains (W and E), emphasizing further the metabolic burden of recombinant mTNFα production in *S. lividans* cells.

### Interpretation of the metabolic profile and footprint

**Fig. 6:**
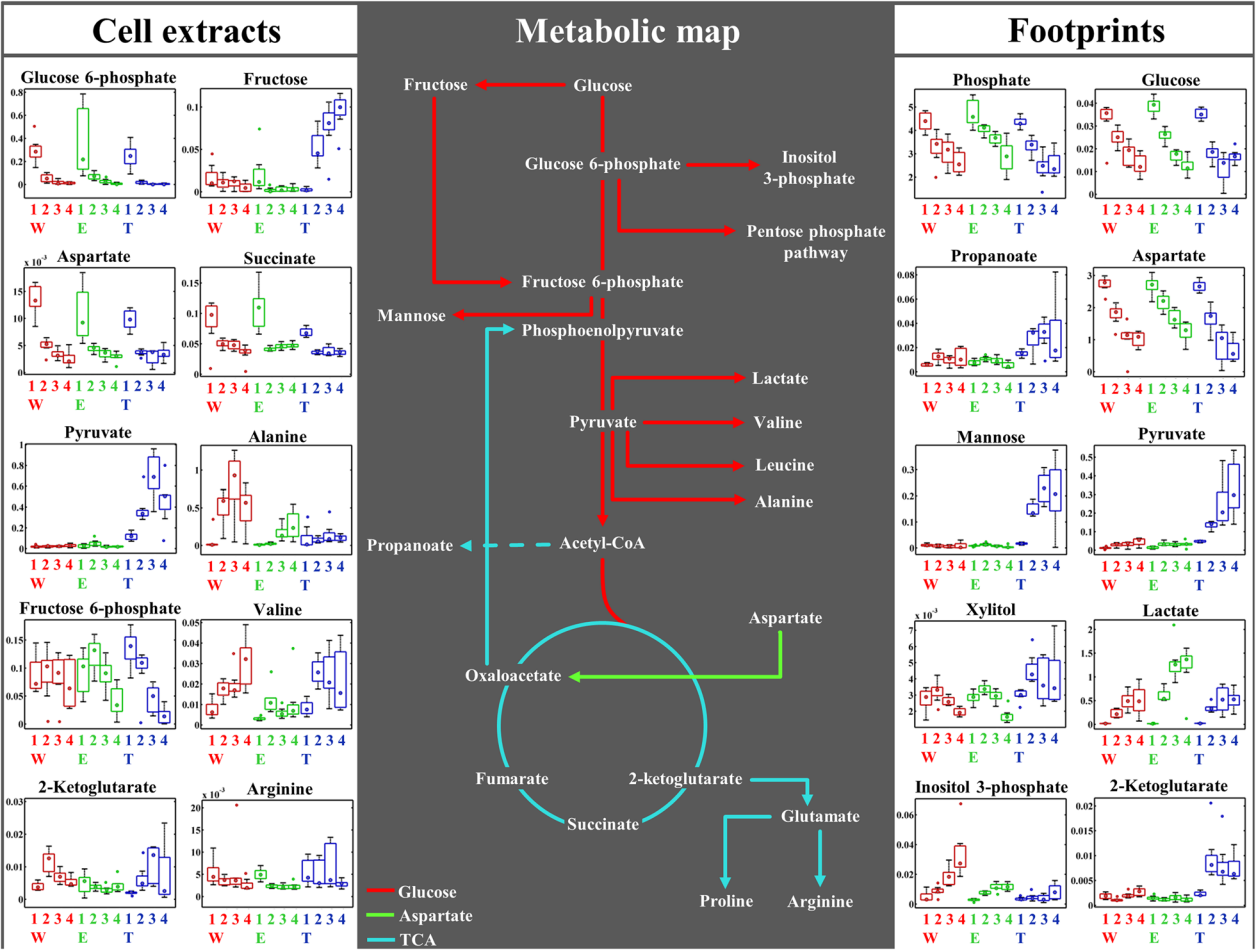
The relative peak areas (*box plots*) of the significant metabolites identified by CPCA-W of cell extracts and footprints GC–MS data are plotted onto the metabolic map of *S. lividans* TK24. *Different colours of the box plots* indicate different strains, wild (*W*, *red*), empty plasmid (*E*, *green*) and mTNFα-producer (*T*, *blue*). While the *numbers 1–4* represent separate sampling time points, 24, 48, 72 and 96 h respectively. *Different coloured arrows* represent the potential contribution of each substrate towards various metabolites and pathways

Block loadings (data not shown) of the different GC–MS data blocks, including metabolic profile (Fig. 4) and footprint data sets (Additional file 1: Figure S2), were employed to identify the most significant metabolites contributing towards the observed clustering patterns. The relative peak intensities of these metabolites at separate time points were plotted as box–whisker plots and overlaid onto the metabolic map of *S. lividans* (Fig. 6), to determine the differences and changes in the metabolic behaviour of the examined strains during the time-course of the experiment. Full lists of these significant metabolites and their corresponding MSI level of identification [46] can be found in the supplementary information (Additional file 1: Tables S1, S2).

The levels of glucose and aspartate in the footprint of all three strains (Fig. 6) demonstrated a gradual depletion, as did aspartate and glucose 6-phosphate (Fig. 6). This is not surprising as *S. lividans* can take up these metabolites and use them as carbon and/or nitrogen sources [43, 44]. However, the footprint data clearly displayed a higher uptake and consumption rate for both aspartate and glucose by mTNFα-producing strain (strain T) compared to non-producing strains (W and E).

The aspartate taken up by the cells is converted to oxaloacetate, via the activity of aspartate oxidase (EC 1.4.3.16), and directed towards the tricarboxylic acid (TCA) pathway [47] (Fig. 6, green arrow). The produced oxaloacetate can then be converted to citrate, by citrate synthase (EC 2.3.3.1) and continue its path in the TCA cycle, providing different precursor metabolites (e.g. 2-ketoglutarate, as well as ATP and NADH) feeding into various amino acids biosynthetic pathways. In addition, oxaloacetate could also be directed towards gluconeogenesis and pyruvate production through the activity of phosphoenolpyruvate carboxykinase (EC 4.1.1.32) (Fig. 6, cyan arrow).

The footprint results (Fig. 6) displayed the excretion or secretion of several organic acids including pyruvate, lactate, 2-ketoglutarate and propanoate. Although *Streptomycetes* species are considered strictly aerobic, accumulation of lactate by these microorganisms in the culture medium has been reported previously [43, 48]. It is commonly believed that due to the unique morphological properties and life cycle of these bacteria, resulting in cell-pellet (clump) formation during growth in liquid culture, oxygen availability could be limited in the centre of the pellets resulting in a micro-aerobic environment in which lactate may be produced through conversion of pyruvate by the activity of lactate dehydrogenase (EC 1.1.1.28) [48, 49]. The lactate excreted by W and T strains during the entire experiment was at comparable levels, while E strain exhibited a slightly higher level (Fig. 6).

Several studies have reported the excretion or secretion of pyruvate and 2-ketoglutarate by different *Streptomyces* species, when grown on defined and complex media containing rapidly usable carbon and nitrogen sources, such as glucose and amino acids (e.g. aspartate) [47, 50–52]. In this study, although the footprint of W and E strains (Fig. 6) also exhibited the accumulation of pyruvate and 2-ketoglutarate in the medium, these metabolites were at much higher levels for the strain (T) producing mTNFα. The detected levels of pyruvate and 2-ketoglutarate in the cell extracts followed the same trend (Fig. 6). These metabolites displayed an almost constant level in the cell extracts of W and E strains throughout the different time points. By contrast, T strain exhibited the accumulation of pyruvate and 2-ketoglutarate (Fig. 6) which started after the first 24 h, coinciding with an increase in mTNFα production (Fig. 1).

Madden and colleagues [47] used fluxomics strategies to trace the contribution of glucose and different amino acids towards the excretion of various organic acids. These authors reported that *S. lividans* cells grown in minimal medium, with glucose and aspartate as carbon and/or nitrogen sources, demonstrated a higher contribution of glucose (21 %) towards the excreted organic acids, than that of aspartate (5 %).

Our results are in agreement with the above studies, as demonstrated by the GC–MS footprint analysis (Fig. 6), although aspartate was found to be at lower levels in T strain compared to W and E strains, the level of this amino acid in the cell extracts of T strain was also lower than that of W and E strains. These findings suggest that T strain utilizes aspartate at a higher level compared to W and E strains. This could be a reflection of the burden of recombinant mTNFα production on cellular metabolism [19, 21], due to higher demand for energy (e.g. NADH and FADH_2_), metabolites and precursors (e.g. amino acids), which can be provided through consumption of aspartate and its contribution towards various TCA intermediate metabolites.

2-ketoglutarate levels displayed complementary results, as this was also higher in T strain compared to W and E strains, whilst the level of succinate in the cell extracts was not significantly different in all three strains. These findings are consistent with the aspartate uptake and utilization trend detected in T strain, which could be explained by the increased rate of TCA pathway resulting from the excess feeding of aspartate into this pathway, in the form of oxaloacetate, which leads to the overflow of 2-ketoglutarate in the cells resulting in a higher excretion rate of this metabolite by T strain in comparison to W and E strains (Fig. 6). As propanoate production is linked to acetyl-CoA, the detected levels of propanoate in the footprint may support this claim, as it displayed much higher levels in T strain compared to W and E strain. This could mean higher acetyl-CoA availability in T strain which may also feed into the TCA cycle.

These observations suggest that although aspartate may contribute towards pyruvate production (as discussed above), its contribution towards the TCA cycle is of higher significance, especially for the T strain. This is perhaps not surprising, considering the role of TCA cycle towards energy production and also contribution of its intermediates towards the biosynthesis of various amino acids (e.g. glutamate, arginine and proline), which could be under high demand in the recombinant mTNF-producing strain, due to the required energy for secretion of mTNFα and/or the production of various proteins involved in the Sec translocon pathway [53].

The arginine levels detected in the cell extracts confirmed the above argument, as it exhibited an increasing trend in the T strain whilst it remained almost unchanged in the W and E strains, throughout the different time points. However, valine accumulated in the cell extracts of W and T strains whilst it remained constant in E strain. Alanine was a special case in this study, as it was accumulated at much higher levels in W strain and also marginally in the E strain compared to T strain (Fig. 6). This could be due to an overflow of this amino acid in the W and E strains, while T strain utilizes alanine at higher rate due to the recombinant mTNFα production. This observation is also not surprising, as alanine is the most prevalent amino acid (13.6 %, Additional file 1: Table S3) found in mTNFα. In addition, alanine production could also serve as an overflow mechanism, by which excess carbon and nitrogen can be removed from the cell by converting pyruvate to alanine via the activity of alanine dehydrogenase (Fig. 6). This may have contributed to lower pyruvate accumulation and higher alanine levels in the W strain, while due to higher demand for nitrogen in the T and E strain, alanine remains at comparably lower levels.

Comparison of the footprint data suggested that glucose was also utilized by T strain at a higher rate compared to W and E strains (Fig. 6). However, the level of glucose 6-phosphate in T strain was lower compared to that of E and W strains (Fig. 6). Fructose 6-phosphate exhibited a similar response, where it was depleted in the T strain at a much higher rate compared to the W and E strain (Fig. 6). This is to be expected as fructose 6-phosphate is positioned between glucose 6-phosphate and pyruvate, which are two of the most highly consumed metabolic substrates feeding into pentose phosphate, amino acid biosynthesis and TCA pathways.

The combined effects of the above pathways could be explained as follows: (1) the redirection of glucose 6-phosphate from glycolysis towards pentose phosphate pathway (Fig. 6) may reduce the flux of carbon feeding into glycolysis, reducing the fructose 6-phosphate production rate; (2) contribution of pyruvate towards the TCA cycle and its consumption by the cells as one of the main substrates for production of various metabolites may accelerate the consumption of fructose 6-phosphate and its flux through its following reactions, depleting the pool of this metabolite.

In addition, the level of fructose detected in the cell extracts of the T strain (Fig. 6) was significantly higher than those of W and E strains, which may possibly be due to the conversion of glucose to fructose via the activity of xylose isomerase (EC 5.3.1.5). The fructose resulting from this reaction could be phosphorylated and converted to fructose 6-phosphate via the activity of fructokinase (EC 2.7.1.4) enzyme. The above pathway could be a stress response mechanism activated by the high demand for fructose 6-phosphate in T strain, acting as a shortcut pathway to replenish the fructose 6-phosphate pool. This could be investigated further in future studies by employing enzyme analysis and metabolic fluxomics strategies.

Mannose, xylitol and inositol 3-phosphate (Fig. 6) were also identified as significant metabolites affecting the clustering pattern in the super scores plots (Fig. 4). Inositol 3-phosphate in *Streptomyces* is produced from glucose 6-phosphate via the activity of myo-inositol-1-phosphate synthase (EC 5.5.1.4), which can then be converted to inositol by inositol-phosphate phosphatase (EC 3.1.3.25) (Fig. 6). Zhang and colleagues [54] reported the importance of inositol in *Streptomyces* during cellular differentiation and growth. Our footprint results demonstrated that, as the biomass of different *Streptomyces* strains increased with incubation time and the cells were passing through different growth phases (Fig. 1), the level of inositol 3-phosphate (Fig. 6) also increased accordingly. However, T strain exhibited the lowest level of this metabolite throughout the different time points, which might be linked to the stress of recombinant mTNFα production, interfering with various developmental processes.

## Conclusion

In this study we observed that recombinant mTNFα production did not significantly affect the final biomass yield or growth rate of the mTNFα-producing strain (Fig. 1), which is perhaps not surprising given the low levels of mTNFα produced (only 18 mg/L). The results obtained from the FT-IR fingerprinting and GC–MS metabolic profile and footprint analyses clearly demonstrated the metabolic effects of mTNFα-production on the streptomycetes cells. An overflow metabolism of several organic acids, including pyruvate, 2-ketoglutarate, and propanoate, was evident in the mTNFα-producing strain which resulted in the excretion of these metabolites by the cells. Several sugars (xylitol, mannose and fructose) were also detected at significantly higher concentrations in the T strain compared to the W and E strains.

Overall the GC–MS results demonstrated a higher uptake and consumption rate of glucose and aspartate by the mTNFα-producing strain compared to the wild type and empty plasmid bearing strains. The above observations could be linked with the imposed metabolic load of recombinant mTNFα production in *S. lividans*, directing available resources towards various essential pathways to meet the metabolite and energy demand resulting from the recombinant mTNFα production and secretion.

Comparison of the growth profile of the mTNF-producing strain with the level of mTNFα produced by this strain at different phases of growth, suggested that although the recombinant mTNFα production is controlled by a constitutive promoter, no direct correlation was found between biomass levels and mTNFα production. These findings put forward the claim that, even though using aspartate as the nitrogen source improved the final biomass yield, it did not increase mTNFα production. This suggests that aspartate is mainly directed towards biomass production and not protein synthesis. However, a clearer picture on the consumption of aspartate and its contribution towards recombinant protein production in *S. lividans* could be achieved by employing fluxomics strategies in future studies. This is of course worth pursuing, as it may provide deeper insights into the metabolic response of *Streptomyces* towards different substrates and the rate to which different metabolic pathways are activated, which may further aid and support the production and optimisation of various recombinant products in this important industrial microorganism.

## Methods

All chemicals were purchased from Sigma Aldrich, UK unless stated otherwise.

### Bacterial strains

*Streptomyces lividans* TK24 was kindly provided by Prof. Lieve Van Mellaert, Rega Institute, KU Leuven, Belgium. The strains used in study include: (1) *S. lividans* wild type (denoted as W), (2) *S.* *lividans* harbouring the plasmid pIJ486 (denoted as E), (3) *S.* *lividans* harbouring pIJ486 encoding and secreting mTNF-α (denoted as T) [43].

### Spore stock preparation

Initial spore stocks for all the strains were prepared by inoculating 25 mL of phage medium (glucose 10 g/L, tryptone 5 g/L, yeast extract 5 g/L, Lab lemco 5 g/L, CaCl_2_·2H_2_O 0.74 g/L, MgSO_4_·7H_2_O 0.5 g/L, containing 50 µg/mL thiostrepton where necessary) with a single colony of each strain, followed by 72 h incubation at 27 °C with 280 rpm shaking using a Multitron standard shaker incubator (INFORS-HT Bottmingen Switzerland). 1 mL of mycelium from each strain was spread-plated onto mannitol soya flour agar (agar 15 g/L, mannitol 20 g/L, soya flour 20 g/L) under sterile conditions, followed by incubation at 27 °C for 7–14 days. Spores were collected from the surface of the agar and washed as described by Kieser et al. [55], resuspended in 1–2 mL sterile 20 % glycerol, briefly agitated and stored at −80 °C.

### Culturing condition

Pre-cultures were prepared by inoculating 50 mL of phage medium, in 250 mL baffled conical flasks, with spore stocks to a final density of 10^8^ spores per mL to promote dispersed growth and avoid clump formation. The flasks were incubated at 30 °C with 280 rpm shaking for 24 h using a Multitron standard shaker incubator. Cells were harvested by centrifugation (4000* g* at 4 °C for 10 min), supernatant was removed and the biomass was washed twice using 50 mL sterile 0.9 % (*w/v*) saline solution.

Washed cells were resuspended in 20 mL of modified minimal medium (NMMP [55] containing: glucose 10 g/L, aspartic acid 13.3 g/L, NaH_2_PO_4_ 2.7 g/L, MgSO_4_·7H_2_O 0.6 g/L, K_2_HPO_4_ 3.92 g/L and 1 mL/L of trace elements solution (ZnSO_4_·7H_2_O 1 g/L, FeSO_4_·7H_2_O 1 g/L, MnCl_2_·4H_2_O 1 g/L, CaCl_2_ anhydrous 1 g/L). The pre-culture suspensions were used for inoculation of the minimal medium (500 mL) with the appropriate strain to a final OD_450nm_ of 0.1. All strains (8 biological replicates of each) were incubated as batch cultures at 27 °C for 96 h with 200 rpm shaking Multitron standard shaker incubator (INFORS-HT, Bottmingen, Switzerland).

### Dry cell weight

To determine the dry cell weight (DCW), samples (2 mL) were taken at different time points (24, 48, 72 and 96 h) and transferred to pre-dried and pre-weighed Eppendorf microcentrifuge tubes (Eppendorf Ltd., Cambridge, UK), followed by centrifugation at 13,000 *g* for 5 min at 4 °C. The supernatant was transferred to a sterile tube to be used for mTNF-α quantification, whilst the cell pellets were washed using sterile distilled water and dried at 55 °C to constant weight.

### mTNFα quantification

Secreted recombinant mTNF-α was quantified using the PeproTech mTNF-α ELISA kit, following manufacturer recommended protocol (PeproTech, Rocky Hill, USA).

### FT-IR analysis

Samples (1 mL) were taken at different time points (48, 72 and 96 h) and the biomass was harvested by centrifugation at 5000 *g*, 4 °C for 5 min. Supernatants were transferred to sterile tubes and flash-frozen in liquid nitrogen to be used for footprint analysis, while the cell pellets were washed twice with 0.9 % saline solution. All samples were stored at −80 °C until further analysis.

Upon analysis, cell pellets were normalized using the saline solution according to their DCW, followed by sonication at maximum power for 1 min to homogenise the cell suspension. All samples were spotted onto an FT-IR silicon plate as 20 µL aliquots and heated to visible dryness at 55 °C. A Bruker Equinox 55 infrared spectrometer (Bruker Ltd., Coventry, UK) was employed for FT-IR spectroscopic analysis of the samples. All FT-IR spectra were collected in absorbance mode in the mid-infrared range (4000–600 cm^−1^) at 4 cm^−1^ resolution, with 64 spectral co-adds following previously published methods [56]. FT-IR data were pre-processed by applying the extended multiplicative signal correction (EMSC) algorithm [57] for scaling of the spectra and to reduce any variation resulting from the sample size, followed by removal of CO_2_ vibrations (2400–2275 cm^−1^) and its replacement with a linear trend.

### Quenching and extraction for GC–MS analysis

Samples (15 mL) taken at different time points were quenched by adding 30 mL of cold (−45 °C) 60 % methanol solution in a 50 mL falcon tube following procedures described previously [58]. Internal metabolites were extracted following a protocol adapted from Ref. [58] with the exception of using 100 % cold (−45 °C) methanol as the extraction solvent and centrifugation speed being 15,871 *g*. All extracts were normalized according to DCW of the samples followed by combining 50 µL from each sample to be used as quality control (QC) [59, 60]. Internal standard solution (0.2 mg mL^−1^ of succinic-*d*_4_ acid, glycine-*d*_5_, benzoic-*d*_5_ acid and lysine-*d*_4_) was added as 100 µL aliquots to all samples before being lyophilised overnight using a speed vacuum concentrator (Concentrator 5301, Eppendorf, Cambridge, UK). Samples were stored at −80 °C until derivatization for GC–MS.

### Derivatization

Derivatization of the samples was carried out via a two-step process: (1) oximation (using methoxyamine-hydrochloride in pyridine), and (2) silylation step [using *N*-Methyl-*N*-(trimethylsilyl) trifluoroacetamide], as described by Fiehn et al. [61] and Wedge et al. [62].

### GC–MS analysis

A Leco Pegasus III mass spectrometer (St Joseph, USA) coupled with an Agilent 6890N GC oven (Wokingham, UK) was employed for the analysis of both footprint and extract samples following previously published methods [63, 64]. All initial identifications adhered to the metabolomics standards initiative (MSI) guidelines [46] followed by removal of mass spectral features within the QC samples with high deviation [62], and normalization of the metabolite peak areas according to the peak area of the internal standard.

### Data analysis

The data collected in this study (FT-IR and GC–MS) were analysed using MATLAB version 9 (The Mathworks Inc., Natwick, USA). All pre-processed FT-IR spectral data were investigated by combining principal component analysis (PCA) [65] with discriminant function analysis (PC-DFA) to reduce the dimensionality of the dataset whilst determining any between-group variation based on a priori knowledge of the experimental class structure [66, 67]. Validation of the PC-DFA was carried out by projection of 12 randomly selected data from each class (test set) onto the resultant PC-DFA model of the remaining replicates (training set), as described elsewhere [68].

All pre-processed and normalized GC–MS peak areas, including those of the metabolites in the media (metabolic footprint) and cell extracts (metabolic profile), were subjected to a form of multiblock PCA [69] known as weighted consensus PCA (CPCA-W) [70]. The CPCA-W functions by arranging the dataset into different blocks of data according to the experimental design and conditions, in order to isolate and therefore study the factor of interest, that is to say it can be used to remove other interfering factors on the data [45, 71]. In this study, the GC–MS data were first arranged into three blocks according to the number of strains, to study the metabolic behaviour of each of the strains individually at the investigated time points (different physiological states). The second approach focused on rearranging the GC–MS data into four blocks based on the separate time points (24, 48, 72 and 96 h) so as to examine the metabolic states of each strain in relation to other strains at each of the individual time points.

## Additional file

10.1186/s12934-015-0350-1 Supplementary file, including the CPCA scores plots of the GC-MS footprint data, and lists of detected metabolites and their MSI level of identification.

## Abbreviations

CPCA-W: consensus principal component analysis-weighted
DCW: dry cell weight
DFA: discriminant function analysis
ELISA: enzyme-linked immunosorbent assay
EMSC: extended multiplicative signal correction
FT-IR: Fourier transform infrared
GC–MS: gas chromatography–mass spectrometry
mTNFα: murine tumour necrosis factor α
NMMP: minimal medium
PCA: principal component analysis
PC-DFA: principal component-discriminant function analysis
QC: quality control
TCA: tricarboxylic acid
TEV: total explained variance
