# Supplementary material for: Metabolomics investigation of recombinant mTNFα production in Streptomyces lividans
Source: Microb Cell Fact. 2015 Oct 9;14:157. doi: 10.1186/s12934-015-0350-1 (PMC4598958; doi:10.1186/s12934-015-0350-1)
Supplement: Supplementary file 1 — 10.1186/s12934-015-0350-1 Supplementary file, including the CPCA scores plots of the GC-MS footprint data, and lists of detected metabolites and their MSI level of identification. [file 12934_2015_350_MOESM1_ESM.docx]

**Supplementary information**

**Metabolomics investigation of recombinant mTNFα production in *Streptomyces lividans***

Howbeer Muhamadali, Yun Xu, David I. Ellis, Drupad K. Trivedi, Nicholas J.W. Rattray, Kristel Bernaerts and Royston Goodacre


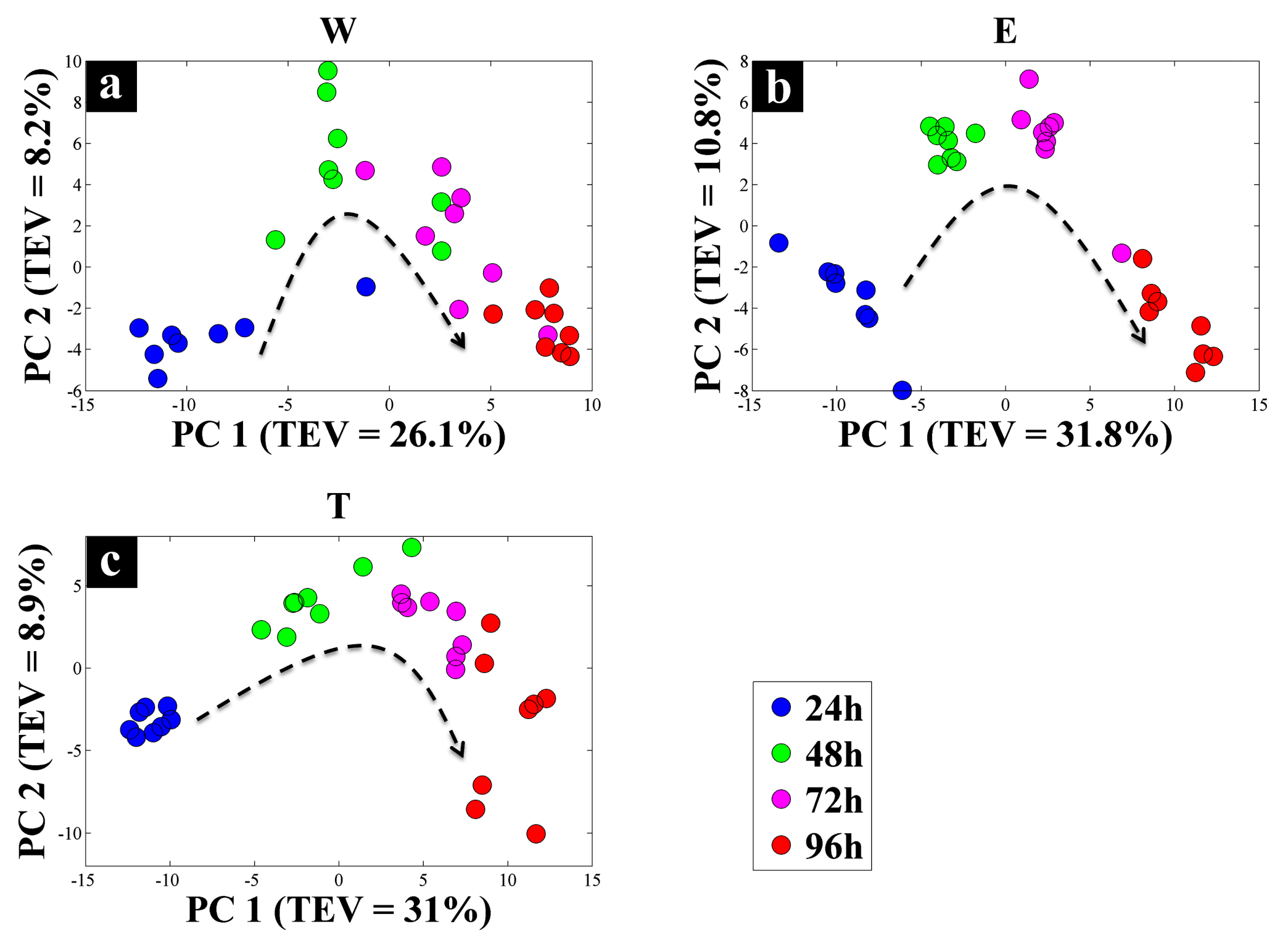


Figure S1. CPCA‑W scores plots of the strain‑blocked GC‑MS metabolic footprint data. The scores plots for each of the strain‑blocked data are presented on a-c plots, where samples taken at separate time points are presented by different coloured circles. The dashed arrows display the direction of the separation according to incubation time. Different alphabets on each plot indicate the *S. lividans* strains; wild (W), empty plasmid (E), and mTNF-producing (T).


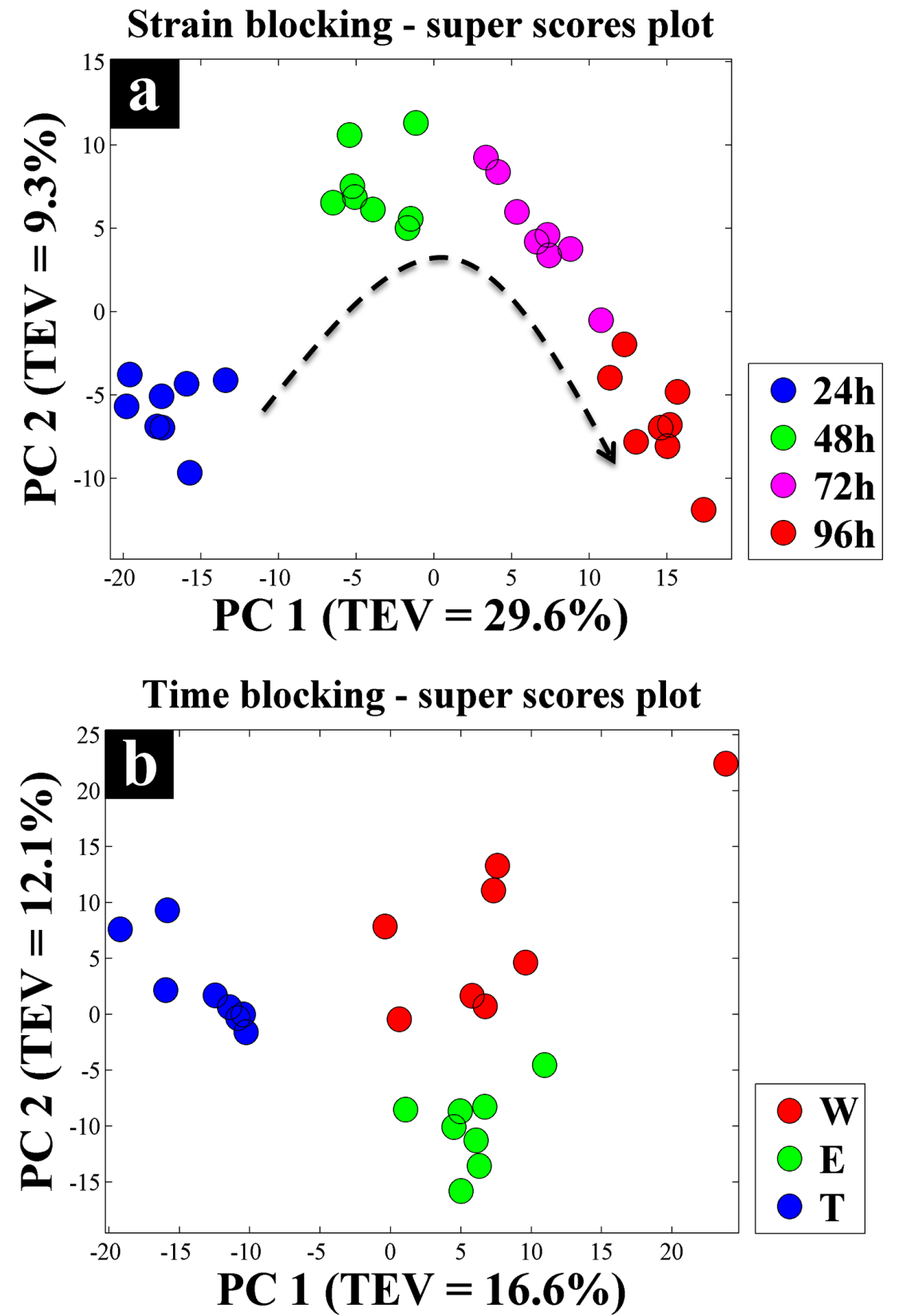


Figure S2. CPCA‑W super scores plots of GC‑MS metabolic footprint data set. (a) Super scores plot of the strain‑blocked model, different coloured symbols indicate sampling time. (b) Super scores plot of the time‑blocked model, where coloured symbols represent different *S. lividans* strains.

**96h**


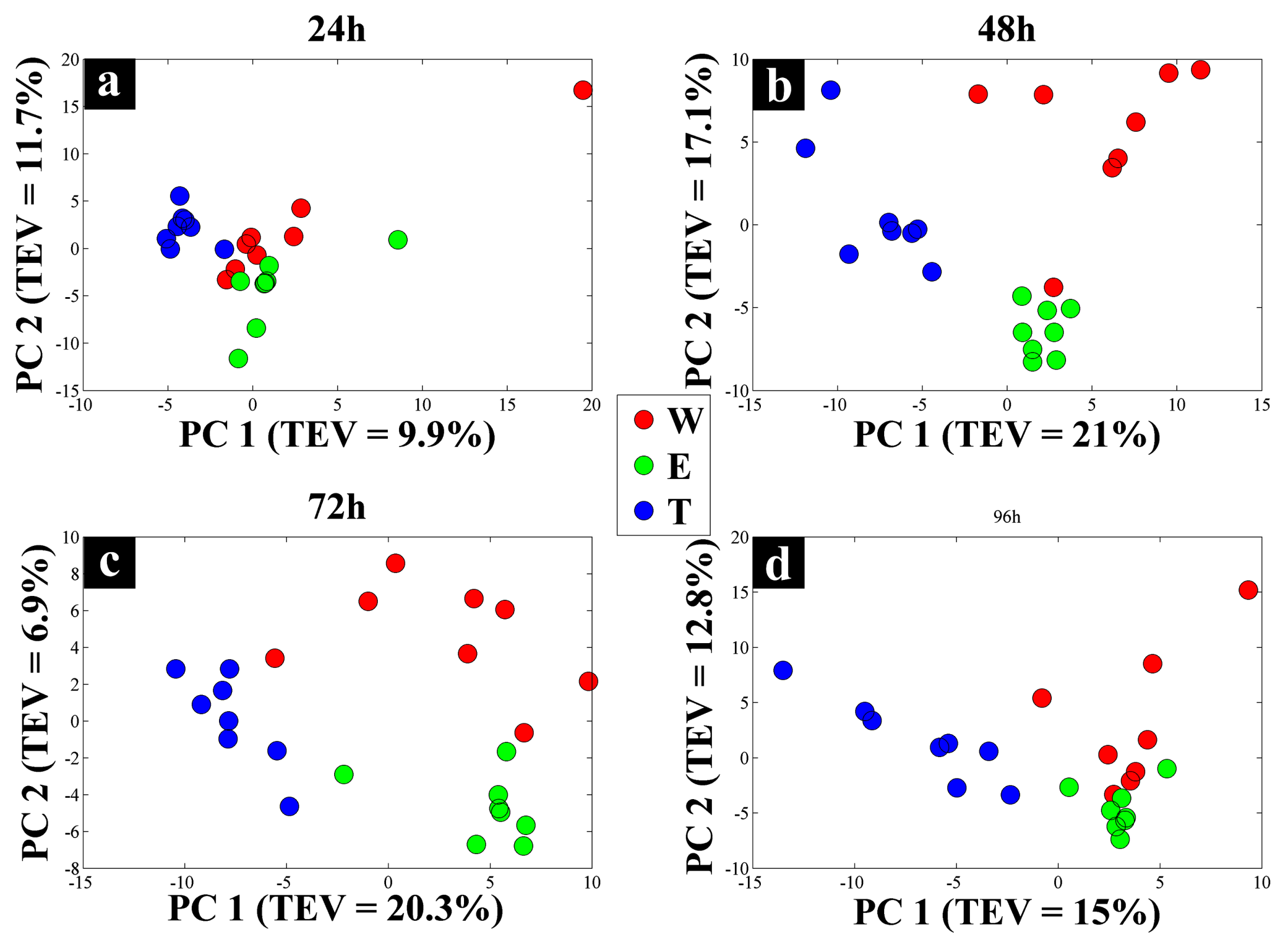


Figure S3. CPCA‑W scores plots of the time‑blocked GC‑MS metabolic footprint data. The scores plots for each of the time‑blocked data are presented on a-d plots, where different coloured circles represent different *S. lividans* strains.


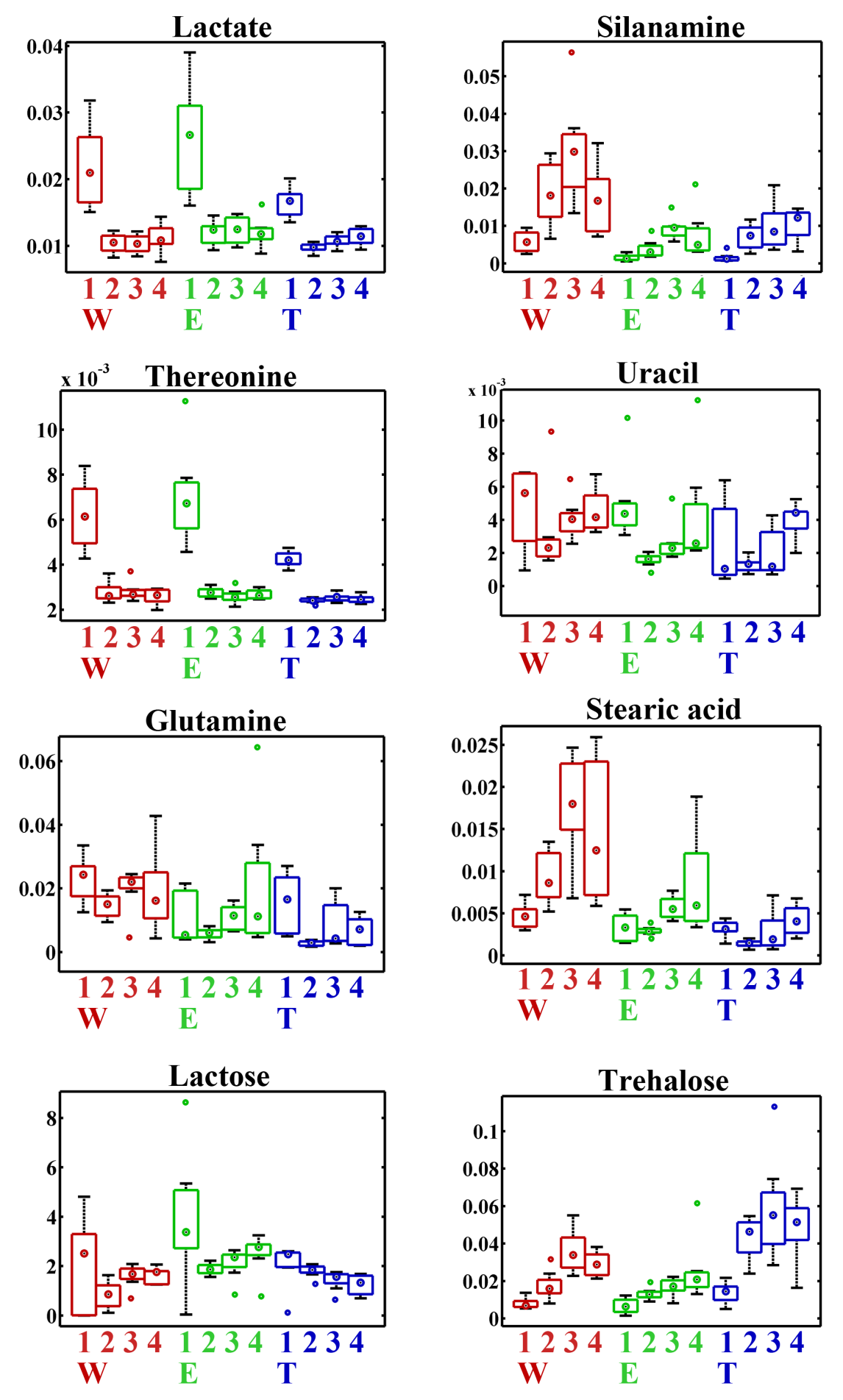


Figure S4. Relative peak area (box whisker plots) of the significant metabolites identified by CPCA-W of cell extract data. Different colours of the box plots indicate different strains, wild (W, red), empty plasmid (E, green) and mTNF-producer (T, blue). While the numbers 1-4 represent separate sampling time points, 24, 48, 72 and 96 h respectively.


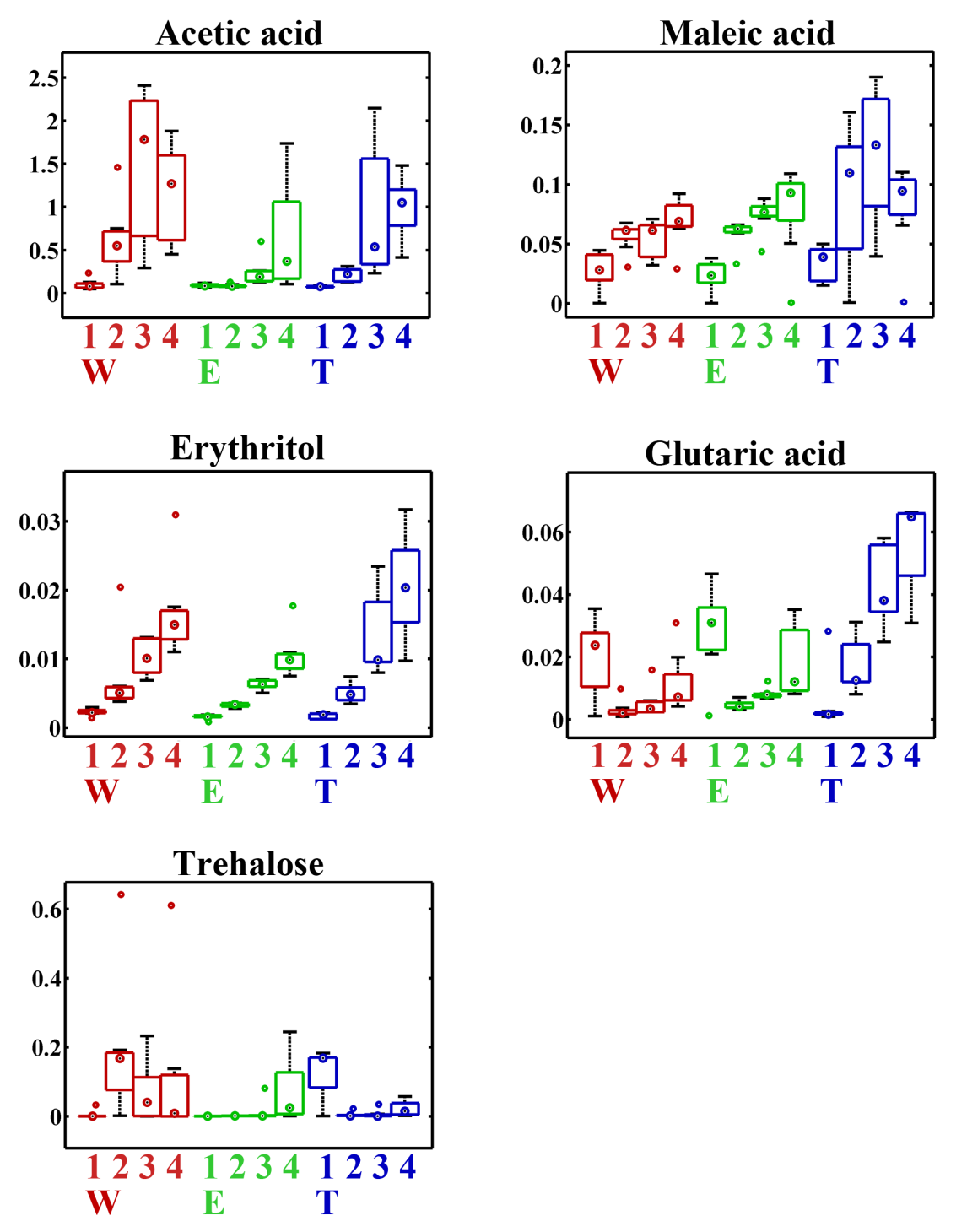


Figure S5. The relative peak area (box whisker plots) of the significant metabolites identified by CPCA-W of footprint data is presented. Different colours of the box plots indicate different strains, wild (W, red), empty plasmid (E, green) and mTNF-producer (T, blue). While the numbers 1-4 represent separate sampling time points, 24, 48, 72 and 96 h respectively.

Table S1. List of the significant metabolites identified by CPCA-W of GC-MS metabolic profile data. All identifications are based on MSI minimum metabolite reporting standards.

| **Variable ID** | **RT** | **RI** | **Metabolite** | **MSI ID level** | **chEBI code** |
| --- | --- | --- | --- | --- | --- |
| 5 | 349.028 | 1040.9 | Lactic acid | 1 | 42111 |
| 9 | 373.928 | 1091.7 | Alanine | 1 | 16977 |
| 14 | 409.978 | 1165.3 | Pyruvic acid | 1 | 32816 |
| 19 | 440.478 | 1227.5 | Valine | 1 | 27266 |
| 21 | 445.878 | 1238.5 | Silanamine | 2 | NA |
| 39 | 520.378 | 1390.6 | Threonine | 1 | 16857 |
| 45 | 530.378 | 1411 | Succinic acid | 1 | 15741 |
| 52 | 556.578 | 1464.4 | Uracil | 1 | 17568 |
| 75 | 614.528 | 1616.6 | Arginine | 1 | 29016 |
| 97 | 652.878 | 1727 | 2-ketoglutaric acid | 1 | 30915 |
| 105 | 660.278 | 1748.3 | Aspartic acid | 1 | 22660 |
| 112 | 666.528 | 1766.3 | Fructose | 1 | 28757 |
| 119 | 685.428 | 1820.7 | Mannose | 1 | 37684 |
| 127 | 700.778 | 1864.8 | Glutamine | 1 | 28300 |
| 164 | 812.028 | 2249.5 | Fructose-6-phosphate | 1 | 78697 |
| 169 | 819.678 | 2276.5 | Stearic acid | 1 | 28842 |
| 170 | 822.378 | 2286 | Glucose-6-phosphate | 1 | 17665 |
| 189 | 885.928 | 2510.3 | Lactose | 1 | 36219 |
| 194 | 902.428 | 2568.6 | Trehalose | 1 | 16551 |

Code: ID, identifier on plots; RT, retention time; RI, retention index; MSI, Metabolomics Standards Initiative identification level; for chEBI codes see: https://www.ebi.ac.uk/chebi/

Table S2. List of the significant metabolites identified by CPCA-W of GC-MS metabolic footprint data. All identifications are based on MSI minimum metabolite reporting standards.

| **Variable ID** | **RT** | **RI** | **Metabolite** | **MSI ID level** | **chEBI code** |
| --- | --- | --- | --- | --- | --- |
| 8 | 353.378 | 1049.8 | Lactic acid | 1 | 42111 |
| 12 | 380.778 | 1105.7 | Propanoic acid | 2 |  |
| 15 | 410.928 | 1167.2 | Pyruvic acid | 1 | 32816 |
| 19 | 436.578 | 1219.5 | Acetic acid | 2 | 15366 |
| 37 | 501.128 | 1351.3 | Phosphate | 1 |  |
| 53 | 530.428 | 1411.1 | Maleic acid | 1 | 18300 |
| 59 | 546.328 | 1443.5 | Erythritol | 1 |  |
| 65 | 568.228 | 1488.2 | Glutaric acid | 1 | 17859 |
| 70 | 586.528 | 1536.1 | Aspartic acid | 1 | 22660 |
| 88 | 616.928 | 1623.5 | Xylitol | 2 | 17151 |
| 105 | 653.128 | 1727.7 | 2-ketoglutaric acid | 1 | 30915 |
| 114 | 675.328 | 1791.6 | Mannose | 2 | 37684 |
| 117 | 684.328 | 1817.5 | Glucose | 1 | 17234 |
| 162 | 827.428 | 2303.9 | Inositol 3-phosphate | 1 | 18169 |
| 197 | 886.578 | 2512.6 | Trehalose | 2 | 16551 |

Table S3. List of relative ratio of amino acid components of mTNFα.

| Amino acids | Number | Total relative ratio % |
| --- | --- | --- |
| Alanine | 25 | 13.4 |
| Leucine | 22 | 11.8 |
| Valine | 19 | 10.2 |
| Serine | 13 | 7.0 |
| Glycine | 12 | 6.5 |
| Glutamine | 12 | 6.5 |
| Glutamic acid | 11 | 5.9 |
| Lysine | 10 | 5.4 |
| Proline | 9 | 4.8 |
| Tyrosine | 8 | 4.3 |
| Aspartic acid | 8 | 4.3 |
| Threonine | 7 | 3.8 |
| Asparagine | 7 | 3.8 |
| Phenylalanine | 5 | 2.7 |
| Arginine | 5 | 2.7 |
| Isoleucine | 3 | 1.6 |
| Cysteine | 3 | 1.6 |
| Histidine | 3 | 1.6 |
| Methionine | 2 | 1.1 |
| Tryptophan | 2 | 1.1 |
